# Harnessing the Biology of Canine Intestinal Organoids to Heighten Understanding of Inflammatory Bowel Disease Pathogenesis and Accelerate Drug Discovery: A One Health Approach

**DOI:** 10.3389/ftox.2021.773953

**Published:** 2021-11-10

**Authors:** Jamie J. Kopper, Chelsea Iennarella-Servantez, Albert E. Jergens, Dipak K. Sahoo, Emilie Guillot, Agnes Bourgois-Mochel, Marilyn N. Martinez, Karin Allenspach, Jonathan P. Mochel

**Affiliations:** ^1^ Veterinary Clinical Sciences, Iowa State University College of Veterinary Medicine, Ames, IA, United States; ^2^ SMART Translational Medicine, Biomedical Sciences, Iowa State University College of Veterinary Medicine, Ames, IA, United States; ^3^ SMART Pharmacology, Biomedical Sciences, Iowa State University College of Veterinary Medicine, Ames, IA, United States; ^4^ 3D Health Solutions, Inc., ISU Research Park, Ames, IA, United States; ^5^ Office of New Animal Drug Evaluation, Center for Veterinary Medicine, Food and Drug Administration, Rockville, MD, United States

**Keywords:** 3D organoids, inflammatory bowel diseases, dietary intervention, one health, dog

## Abstract

In a recent issue of the Lancet, the prevalence of Inflammatory Bowel Disease (IBD) was estimated at 7 million worldwide. Overall, the burden of IBD is rising globally, with direct and indirect healthcare costs ranging between $14.6 and $31.6 billion in the U.S. alone in 2014. There is currently no cure for IBD, and up to 40% of patients do not respond to medical therapy. Although the exact determinants of the disease pathophysiology remain unknown, the prevailing hypothesis involves complex interplay among host genetics, the intestinal microenvironment (primarily bacteria and dietary constituents), and the mucosal immune system. Importantly, multiple chronic diseases leading to high morbidity and mortality in modern western societies, including type II diabetes, IBD and colorectal cancer, have epidemiologically been linked to the consumption of high-calorie, low-fiber, high monosaccharide, and high-fat diets (HFD). More specifically, data from our laboratory and others have shown that repeated consumption of HFD triggers dysbiotic changes of the gut microbiome concomitant with a state of chronic intestinal inflammation and increased intestinal permeability. However, progress in our understanding of the effect of dietary interventions on IBD pathogenesis has been hampered by a lack of relevant animal models. Additionally, current *in vitro* cell culture systems are unable to emulate the *in vivo* interplay between the gut microbiome and the intestinal epithelium in a realistic and translatable way. There remains, therefore, a critical need to develop translatable *in vitro* and *in vivo* models that faithfully recapitulate human gut-specific physiological functions to facilitate detailed mechanistic studies on the impact of dietary interventions on gut homeostasis. While the study of murine models has been pivotal in advancing genetic and cellular discoveries, these animal systems often lack key clinical signs and temporal pathological changes representative of IBD. Specifically, some limitations of the mouse model are associated with the use of genetic knockouts to induce immune deficiency and disease. This is vastly different from the natural course of IBD developing in immunologically competent hosts, as is the case in humans and dogs. Noteworthily, abundant literature suggests that canine and human IBD share common clinical and molecular features, such that preclinical studies in dogs with naturally occurring IBD present an opportunity to further our understanding on disease pathogenesis and streamline the development of new therapeutic strategies. Using a stepwise approach, *in vitro* mechanistic studies investigating the contribution of dietary interventions to chronic intestinal inflammation and “gut leakiness” could be performed in intestinal organoids and organoid derived monolayers. The biologic potential of organoids stems from the method’s ability to harness hard-wired cellular programming such that the complexity of the disease background can be reflected more accurately. Likewise, the effect of therapeutic drug candidates could be evaluated in organoids prior to longitudinal studies in dog and human patients with IBD. In this review, we will discuss the value (and limitations) of intestinal organoids derived from a spontaneous animal disease model of IBD (i.e., the dog), and how it can heighten understanding of the interplay between dietary interventions, the gut microbiota and intestinal inflammation. We will also review how intestinal organoids could be used to streamline the preclinical development of therapeutic drug candidates for IBD patients and their best four-legged friends.

## Inflammatory Bowel Disease–A Multifactorial Disease

Inflammatory Bowel Disease (IBD) is a serious chronic relapsing inflammatory disorder that primarily affects the gastrointestinal (GI) tract. IBD affects over 2 million adults in the United States (US), 2.5–3 million in the European Union (EU) and 7 million worldwide, with the prevalence of IBD consistently increasing over time (Bonen and Cho, 2003; Burisch et al., 2013; GBD 2017 Inflammatory Bowel Disease Collaborators, 2020; Ng et al., 2017). IBD detrimentally impacts the psychological and physical quality of life of patients through GI symptoms, health care costs, extra-intestinal manifestations, and interference with employment, education, and proper nutrition (Knowles et al., 2018). New epidemiological data suggest that the incidence and prevalence of the diseases are increasing, and medical therapy and disease management have changed significantly in the last decade. The economic impact of IBD is also substantial with direct and indirect healthcare costs ranging between $14.6 and $31.6 billion in the U.S. alone in 2014 and direct healthcare costs of 4.6–5.6 bn Euros/year in the EU (Burisch et al., 2013; Alatab et al., 2020). Thus, improved treatments are desperately needed.

**FIGURE 1:**
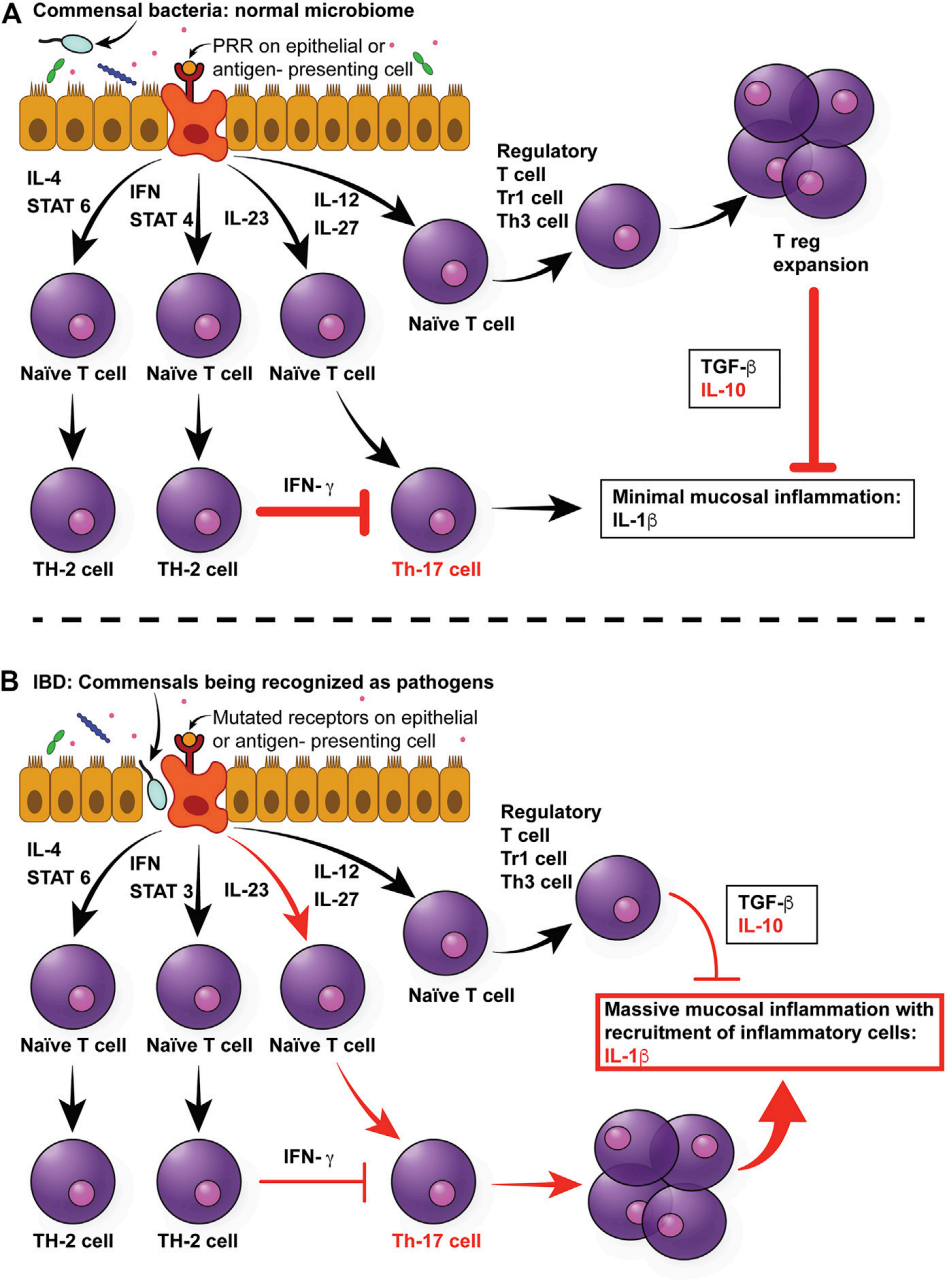
Multifactorial pathophysiology of canine IBD. **(A)** In the normal intestinal mucosa, Toll-like receptors (TLRs) sample pathogen-associated molecular patterns (PAMPs) from commensals in the intestinal lumen, which send signals to naïve T cells to differentiate primarily into T regulatory cells, which produce anti-inflammatory cytokines, such as TGF-beta and IL-10. **(B)** In the case of canine IBD, microbial dysbiosis drives the messaging toward a pro-inflammatory pathway of Th cell differentiation, resulting in the production of pro-inflammatory cytokines, mainly IL-1beta. In addition, mutations in pattern recognition receptors, such as TLR5, result in hyper-responsiveness to flagellin. Since the dysbiosis in canine IBD is characterized by an increase in *Enterobacteriaceae* (which express flagellin), this will further increase pro-inflammatory responses of the mucosa. Moreover, the inflammatory cytokines will lead to architectural changes in epithelial cells, such as increased leakage through tight junction, and therefore increased permeability. This in turn will result in more bacteria breaching the mucosal barrier, therefore leading to a self-enhancing circle of inflammation. IL-4: Interleukin 4, IFN: Interferon, STAT3: Signal Transducer And Activator Of Transcription 3, IL-23: Interleukin-23, IL-12: Interlleukin-12, IL-27: Interleukin-27, TGF-beta: Tissue growth factor-beta, IL-10: Interleukin-10, IL-beta: Interleukin-beta.

IBD is a multifactorial disease with a complex pathogenesis related to interplay between genetic predisposition and a multitude of environmental triggers that, collectively, negatively impacts intestinal microbiota, epithelial permeability, and ultimately, leads to inappropriate intestinal immune activation as shown in Figure 1 (Piovezani Ramos and Papdakis, 2019). IBD in humans consists of two broad yet distinctive clinical and histopathologic phenotypes, Crohn’s disease (CD) and Ulcerative Colitis (UC). CD can affect any portion of the intestinal tract, but the terminal ileum is most frequently implicated with a transmural, discontinuous pattern of lesions. In contrast, UC lesions are localized to the mucosa and submucosa with a continuous pattern that primarily affects the colon and proximal rectum although secondary inflammation of the adjacent terminal ileum may occur due to proximity (Fakhoury et al., 2014). The multifactorial nature of IBD has made investigations into disease pathogenesis as well as development of treatment modalities challenging.

Unfortunately, IBD is not a single gene disorder, but rather has a complex genetic component (Graham and Xavier 2020). GWAS and meta-analyses of 25,305 IBD cases identified approximately 300 risk loci for developing IBD (De Lang et al., 2017). Trans-ancestry studies of IBD identified differential risk associations for *NOD2* being more prevalent in European populations and *TNFSF15* being more prevalent in East Asian populations (Liu et al., 2015). Twin studies have underscored that while there is a genetic contribution to IBD, the disease is not inherited in a simple Mendelian fashion (Farmer et al., 1980; Monsen, et al., 1987; Halme et al., 2006). In fact, genetic linkage studies have identified multiple susceptibility genes such as CARD15/NOD (Hugot, 2004), autophagy genes ATG16L1 and IRGM (Glocker et al., 2009; Noguchi et al., 2009). And, specifically in young children with IBD, IL-10R polymorphisms have been described and characterized (Moran et al., 2013; Shim et al., 2013). Noteworthily, the variable contribution of genetic susceptibilities and associated genes is one contributing factor to the lack of novel successful therapeutic and diagnostic modalities.

IBD and other autoimmune disorders occur at a lower incidence in less industrialized nations. One possible explanation for this lies in the “hygiene hypothesis” which proposes that several factors affecting early life environments including exposure to common infectious agents and use of antimicrobials is necessary to program the immune system for appropriate future responses to antigenic stimulation (Koloski et al., 2008). Early exposure to microbes helps establish an important balance between pro-inflammatory Th1 helper type 1 (Th1) cells responses and tolerant regulatory (Treg) responses. These early exposures may help prevent hyperactive immune responses to intestinal microbiota and other stimuli. Conversely, overly clean environments may contribute to an untrained and therefore exaggerated immune response when antigens are encountered later in life (Strachan, 1989; Hoshen et al., 2008; Kondrashova et al., 2013).

In addition to, and as part of early life exposures, the host GI microbiota not only play a crucial role in maintaining normal GI physiology but also have been implicated as having a key role in the development of IBD (Cho, 2008). In fact, in some individuals multiple bacterial, viral, fungal and parasitic infections have been implicated to increase the risk of developing IBD as well as contribute to relapse in patients with previously well controlled disease (Axelrad et al., 2021). While pathogen exposure or “pathogen trigger” is believed to contribute to the development of IBD in some individuals (Axelrad et al., 2021), a unique and distinct pathogen has not been associated with all cases of IBD. Overall, the so-called “pathogen trigger” hypothesis may be related to alterations in the intestinal microbial community that contribute to the development of IBD in susceptible individuals.

Although IBD was initially thought to be due to specific pathogen exposure, current studies propose that perturbations in commensal enteric bacteria play a role in the development of IBD (Packey and Sartor., 2008). Commensal GI microbiota may contribute to the development of IBD by carrying atypical virulence factors or constituting abnormal compositions of the microbiota resulting in an altered metabolome (Bourgonje et al., 2021). On the other hand, it has been speculated that if the host has defective intestinal barrier function resulting in increased immune response against commensals. Several studies have reported a decrease in GI microbial diversity in IBD patients (Frank et al., 2007; Scanlan and Marchesi, 2008), where the composition and diversity of beneficial microbial community members is reduced while the numbers of potentially harmful bacteria (such as *Enterobacteriaceae*) are increased in patients with IBD (Scanlan et al., 2006). Specific bacteria and/or virulence factors that lead to the development of IBD in an individual have not yet been identified; however various candidates have been suggested. For example, changes in populations of resident *Escherichia coli* have been recorded in patients with CD (Frank et al., 2007), including increased antibody titers to the *E. coli* outer membrane protein C (OMP C) in patients with IBD (Arnott et al., 2004). Similarly, in one study, the presence of adherent invasive *E. coli* was found in 65% of ileal resections in chronic inflammation (Darfeuille-Michaud et al., 2004). The increased incidence of microbial fluctuations may contribute to a heightened immune response to what would otherwise be considered normal GI microbial components (Reiff and Kelly, 2009).

In addition to alterations in GI microbial community members and composition, the metabolome, or metabolic products of the resident GI microbial community may contribute to the development of IBD (Roediger et al., 1993). For example, butyrate, a metabolic byproduct of beneficial microbiota (*Clostridiales*, *Bacteroidetes*), is a source of energy for colonic epithelial cells which improves the GI epithelial barrier integrity and the host immune response (Roediger et al., 1993). Increases in sulphate-reducing bacteria have also been recorded in IBD patients; sulphate-reducing bacterial species produce hydrogen sulfide, and their presence has been linked to blockage of butyrate use by colonocytes (Christl et al., 1996; Thibault et al., 2010). The functional composition of a microbial community has gained interest in recent years as opposed to simply the presence/absence or relative abundance of specific microbial community members.

Furthermore, some individuals with IBD appear to have reduced immune tolerance to their normal GI microbiota (Cho, 2008; Yap and Marino, 2018). In normal individuals, the immune system elicits appropriate anti-microbial responses against pathogens while tolerating the host commensal GI microbiota. The underlying basis for this interaction is a defective or amplified cross-communication between the GI microbiota and the host’s immune system. Specifically, four potential defective microbiota-immune system interactions have been hypothesized to contribute to IBD pathogenesis (Figure 1) (Packey and Sartor, 2008). In the first and second scenario, an increase in the abundance of pathogenic bacteria, or virulence of the normal microbiota may cause increased stimulation of both adaptive and innate immune responses. In the third scenario, an altered, although non-pathogenic composition of the GI microbiota can adversely affect the GI physiology, making mucosal surfaces more susceptible to damage and invasion. In the fourth and last scenario, even with normal populations of commensal bacteria, the host’s ability to prevent bacteria from crossing the mucosal barrier may be impaired. A key role of altered or decreased intestinal barrier function has been demonstrated in both human and animal models of IBD (Kobayashi et al., 2007; Gerova et al., 2011; Dorofeyev et al., 2013; van der Post et al., 2020). Further, recent evidence has emerged to suggest that epithelial barrier dysfunction is a primary defect of IBD, rather than a secondary consequence to inflammation, due to mutations in genes regulating intestinal epithelial cell function (Keita et al., 2018).

An individual diet can have both direct and indirect effects on the microbial community, metabolome, intestinal barrier function and intestinal mucosal immunity crosstalk (Gasaly et al., 2021). Globally, the consumption of highly processed diets has been positively associated with an increased risk of IBD development (Narula et al., 2021). Specifically, a high protein, high fat, low fiber diet, commonly referred to as a “westernized diet”, has been shown to adversely affect intestinal permeability (Tanka et al., 2020), the metabolome (David et al., 2014), GI mucosal microbial communities and pathogen susceptibility (Desai et al., 2016), all of which play key roles in the development of IBD.

In summary, IBD represents a spectrum of serious GI diseases due to a multitude of contributing factors which may vary between patients and patient populations.

## Pitfalls of Current IBD Models–A Roadblock in Treatment Discovery

### Challenges With Traditional Rodent Models of IBD

A 2003 review identified 63 different animal models of IBD within the scientific literature (Hoffman et al., 2003). These animal models were classified within one or more of the following categories: 1) antigen-specific and bacterial models; 2) other inducible models (chemical, immunological, and physical); 3) genetic models (transgenic and knockout); 4) adoptive transfer models; and 5) spontaneous animal models. Most rodent models used for the study of IBD are the result of one or two defined factors such as genetic manipulations (e.g., IL-10 deletions/knockouts), GI irritants (e.g., dextran sulfate sodium) and/or pathogen exposure (e.g., *Helicobacter hepaticus*)–true spontaneous rodent models do not exist as they do not develop IBD spontaneously. Furthermore, rodent models fail to adequately recapitulate many of the factors suspected to play a role in the development of IBD in some humans including early life exposures and the role of select dietary constituents. Although the use of inducible, genetic and/or transfer models provides pathophysiological insight for certain aspects of IBD, the inability of these models to fully emulate the multifactorial nature of IBD results in a high rate of pharmaceutical and treatment failures when candidates are first tested in mice and then brought forward to human clinical trials (Hackam and Redelmeier, 2006). The continued use of models that have failed to accurately predict human clinical responses not only delays identification of treatments that are desperately needed but also wastes valuable time and resources.

Ideally, a disease animal model should closely mimic the natural population. Yet, the multifactorial nature of IBD significantly limits the usefulness and translatability of traditional rodent models to study and elucidate treatment modalities for IBD as outlined in Table 1 (DeVoss and Diehl, 2014; Oh et al., 2014). Also, while many rodent models of IBD exist, none adequately predict human response to candidate treatments (Valatas et al., 2013; Pizarro et al., 2019). Thus, results from these models have limited translatability for predicting treatment response and safety of candidate drugs to the human population. Conversely, spontaneous animal models are considered to have high clinical and physiological relevance as they more closely mirror the natural course of IBD pathogenesis and chronicity of the intestinal inflammation. Thus, the use of models that represent the true complexity of the disease is imperative to identify and develop new candidate treatments for IBD (Pizarro et al., 2019).

**TABLE 1:** Comparison of human IBD with cIBD in dogs and traditional rodent models of IBD.

Feature	Humans	Canines	Rodents
Genetic basis	Yes	Yes	Engineered
Etiology	Multifactorial	Multifactorial	+/- multifactorial
Intact immune system	Yes	Yes	+/-
Role of GI microbiota	Yes	Yes	Yes
Blood in stool	Yes	Yes	Yes
Diarrhea	Yes	Yes	Yes
Definitive diagnosis	GI mucosal biopsy	GI mucosal biopsy	GI mucosal biopsy
Longitudinal studies	Yes–endoscopy, histology	Yes–endoscopy, histology	No
IBD treatment	Diet + drugs	Diet + drugs	Drugs
Disease heterogeneity	Yes	Yes	Variable

### A One Health Approach to Improving Treatment for IBD–In Human and Canine Patients

The one health initiative highlights the benefits of a multidisciplinary approach to human and animal health issues. The need for large animal models to improve translational science has been widely emphasized by the National Institute of Health (National Institute of Diabetes and Digestive and Kidney Diseases, 2009; NIH symposium, 2012). Dogs represent an underutilized model for human diseases with many appealing attributes, particularly for the study of multifactorial complex diseases such as IBD. The use of dogs with canine IBD (cIBD) presents a unique opportunity for additional data acquisition as well as performing clinical trials in patients suffering from this spontaneously occurring disease. With domestication, canine intestinal anatomy, physiology, and diet have gradually evolved with and mirrored those of their owners (Lyu et al., 2018; Alessandri et al., 2019). Perhaps not surprisingly, with dogs and humans living side-by-side, there is notably more taxonomic and functional overlap of the GI microbiome in humans and dogs (60%) compared to pigs (33%) and mice (10–20%) (Coelho et al., 2018). Comparison of the functional and structural changes of wild versus domesticated dogs support the evolution of domesticated canine GI microbiota to match human GI microbiota which likely has been influenced by a large degree by diet overlap (Lyu et al., 2018). The significant overlap in functional and taxonomic GI microbiota is particularly significant given the role that the GI microbiota, directly or indirectly, play in the majority of contributing factors leading to the development of IBD. Canine IBD refers to a naturally occurring group of chronic idiopathic enteropathies resulting in persistent and/or recurrent GI clinical symptoms (Jergens, 1999; Allenspach et al., 2006; Cerquetella et al., 2010; Simpson and Jergens, 2011; Allenspach et al., 2016). Comparisons of IBD in dogs and humans reveal clinical, genetic, microbial, and pathophysiological similarity between species with few notable exceptions (Xenoulis et al., 2008; Cerquetella et al., 2010; Kathrani et al., 2014; Vázquez-Baeza et al., 2016; Cabrera-García et al., 2020). The true prevalence of cIBD is unknown, however cIBD is the most common histopathologic diagnosis in dogs with chronic GI clinical signs (Jergens, 1999; Allenspach et al., 2016). Like IBD in humans, cIBD is a multifactorial disease which includes a combination of genetic predispositions, alterations in intestinal microbiota, and abnormal intestinal mucosal immune responses (Jergens and Zoran, 2005). The multifactorial contributing factors for the development of IBD/cIBD in humans and dogs, have striking similarities. For example, as with humans, genetic susceptibilities are also suspected to play a role in cIBD given that there are several noted breed predispositions (Breitschwerd, 1992; Churcher and Watson, 1997; German et al., 2000; Littman et al., 2000; Hostutler et al., 2004; Kathrani et al., 2010; Kathrani et al., 2011a; Kathrani et al., 2014). Similar to humans, allelic variation in TLR 2, 4 and 5 have been identified in German Shepard Dogs with cIBD, which contributes to an abnormal response to intestinal microbiota (Allenspach et al., 2010; Kathrani et al., 2012; Kathrani et al., 2014). Additionally, multiple SNPs were identified in NOD2 of dogs with cIBD (Kathrani et al., 2014). Furthermore, similarities in alterations of the intestinal microbiota have been reported between humans with IBD and dogs with cIBD (Simpson et al., 2006; Baumgart et al., 2007). Similarities have also been identified in altered immune responses between humans and dogs with IBD/cIBD. Such abnormalities include a large number of IgE positive cells in dogs with cIBD (Locher et al., 2001), a decrease in mast cell numbers with a concomitant increase in CD3+ cells, IgG+ plasma cells, CD11c cells and reduced Treg cells (Jergens et al., 1996; Jergens et al., 1998; German et al., 2001; Kathrani et al., 2011b; Schmitz et al., 2012). Furthermore, diet has also been shown to impact mucosal microbiota and the GI metabolome and mucosal microbiota in dogs with cIBD (Atherly et al., 2019; Ambrosini et al., 2020a). Recently, early-life risk factors for developing IBD have been an area of interest and studies have identified similar findings in dogs and humans with cIBD/IBD such as an association with early life high-fat low-carbohydrate diets and the later development of IBD/cIBD (Hemida et al., 2021). Like humans, a diagnosis of cIBD is made based on chronic GI signs, eliminating other known causes of intestinal inflammation and histopathologic confirmation of intestinal inflammation. Treatment for cIBD involves sequential treatment with elimination diet, prebiotics and probiotics, and immunosuppressive medications with some dogs failing to respond to currently available treatment options. Overall, many similarities exist between dogs and humans with cIBD and IBD making them an ideal, yet underutilized, spontaneous disease model. Given the high prevalence of cIBD in dogs, utilizing these canine patients, will further IBD research without requiring induction of disease or development of artificial models such as traditionally used rodent knockouts as previously described. Utilizing more accurate spontaneous disease animal models will hopefully improve the translatability and success of pre-clinical trials in IBD research. Subscribing to the one-health approach, use of dogs with cIBD to further IBD research provides an opportunity to improve efficacy and translatability of IBD research which will benefit human and canine patients alike.

**FIGURE 2:**
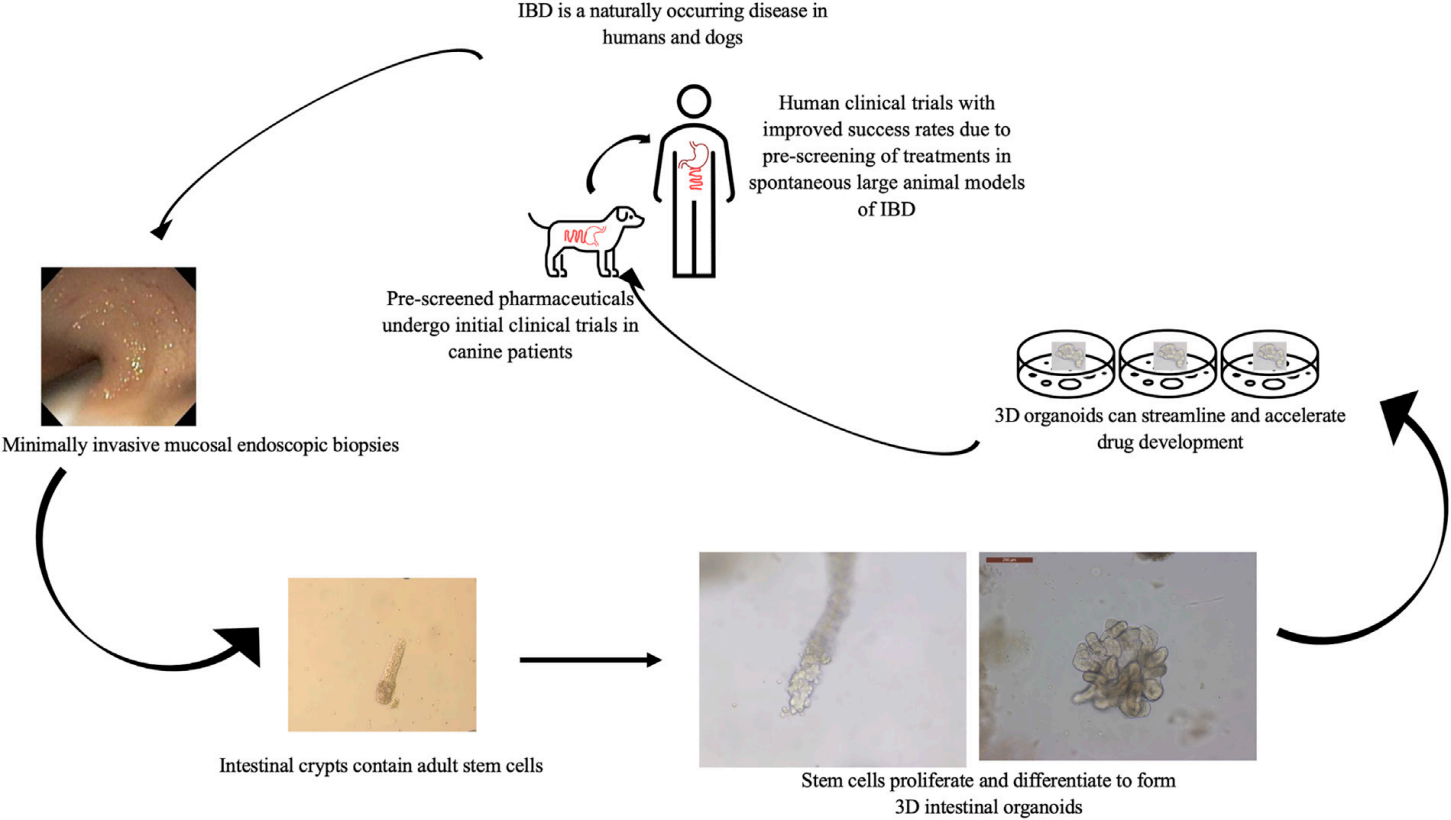
Value of the combined *in vitro*–*in vivo* approach using 3D intestinal organoids and clinical trials in canine IBD patients to streamline drug research and development. Using canines with spontaneously occurring cIBD allows for minimally invasive acquisition of endoscopic biopsies to generate *in vitro* organoid cell culture. Organoids can be used to screen candidate treatments which then can be further evaluated in live canine patients before moving forward with human clinical trials to expedite and improve research efficiency.

Finally, although dogs with cIBD have many attractive attributes for the study of IBD there are several disadvantages including increased cost compared to rodent models and increased welfare concerns given the human-canine bond all of which impede discovery and preclinical testing of candidate treatments for IBD. Thus, recent establishment of canine intestinal organoid systems serve to further one health initiatives (Mochel et al., 2017; Chandra et al., 2019; Kramer et al., 2020) and presents a unique complementary opportunity to advance IBD research. Although the use of canine organoids will not replace the need for live animal models in preclinical testing of candidate therapies for IBD, early use of the organoid technology will accelerate early testing and identification of candidate therapies with an increased probability of success in subsequent live animal and human clinical trials (Figure 2).

## Organoids–A Possible Solution for Translational Research in IBD

With the prevalence of GI diseases (including but not limited to IBD) on the rise, there is a critical need for *in vitro* modeling systems that offer high throughput, reproducibility, and clinical application. Patient-derived organoids have provided substantial advancement in personalized medicine with promising benefits for clinical decision making (Li et al., 2020; Liu et al., 2021). As such, there is strong evidence to support the use of intestinal organoids to fill this critical need for high throughout, reproducible and clinically relevant *in vitro* models (Fair et al., 2018) and, in these efforts, dogs may serve to bridge the gap between basic science and clinical research to allow for the advancement of one health initiatives (Mochel et al., 2017; Chandra et al., 2019).

Organoids can be defined as “cells growing in a defined three-dimensional (3D) environment *in vitro* to form mini-clusters of cells (aka, miniguts) that self-organize and differentiate into epithelial cell types, recapitulating the structure and function of an organ *in vivo* derived from either embryonic stem cells, induced pluripotent stem cells, or adult stem cells” (Corró et al., 2020). For purposes of this review, the term organoid refers exclusively to those cultured from adult stem cells as they offer the greatest utility in translational clinical research. Culture and characterization of intestinal organoids derived from single Lgr5+ adult stem cells in the intestinal crypts were first described in mice (Sato et al., 2009). Notably, these cultures offered a significant advantage over traditional monocultures as they more accurately recapitulated *in vivo* cellular and architectural heterogeneity (Sato et al., 2009). Since their inception, intestinal organoids have been cultured from a wide variety of mammalian species, including gut tissues obtained from humans (Sato et al., 2011), mice (de Lau et al., 2012), dogs (Chandra et al., 2019; Kramer et al., 2020), cats, and several livestock species (Powell and Behnke, 2017). Importantly, intestinal organoid cultures recapitulate cellular heterogeneity, morphological changes, and the polarization of the colonic epithelium with the apical and basolateral sides oriented toward and outward from the lumen, respectively (d’Aldebert et al., 2020). Further, techniques for reproducibly culturing patient-derived organoids have been established (VanDussen et al., 2015).

Intestinal organoid systems are a highly efficient *in vitro* modeling technique that lends itself to high-throughput systems. Creation of cell cultures starts with a collection of intestinal biopsies from human- or veterinary-patients and subsequent isolation of the intestinal stem cells. The use of endoscopically obtained biopsy specimens decreases experimental related morbidity and mortality, ultimately improving welfare and improving experimental efficiency and decreases inter-experimental variation by allowing the same animal to be used multiple times. Within 10–12 days of culture, organoids are highly differentiated from both a phenotypic and morphological aspect (Fair et al., 2018). Recent advancements in biotechnology have further improved the isolation efficiency as well as culture of single intestinal stem cells in manners amenable for high-throughput applications (Gracz et al., 2015). Additionally, Williamson et al. (2018) have developed and validated a high-throughput organoid microinjection system for the study of GI microbiota and luminal physiology. In their study, a microinjection system was used to introduce 0.2 nL of bacterial inoculate into the luminal space of each organoid at a rate of approximately 90 organoids/h, with approximately 500 organoids retaining the bacterial inoculum after an 18 h period. Further, this study showed that even mixed bacterial populations in the inoculate were able to grow within the organoid lumen, that bacterial composition remained stable over a four-day period, and that organoids were unaffected by antibiotics placed in culture media to prevent the contamination of cultures. However, the authors reported that the efficiency of microinjections was highly variable between organoids of different sizes, shapes, or luminal volumes; and, as such, should be optimized further (Williamson et al., 2018). Regardless, this technique holds promise in facilitating the investigation of luminal-microbiota interactions in intestinal organoid cultures. Technology to allow co-culture of organoids and intestinal microbiome is under development. Overall, continued development and optimization of high-throughput methodologies for intestinal organoid culture will be fundamental for utilization in large-scale, clinical studies for IBD or other related chronic intestinal diseases.

As previously mentioned, intestinal permeability appears to play an integral role in the multifactorial nature of IBD. Unfortunately, *in vivo* assessment of intestinal permeability presents several logistical and functional difficulties from both an experimental and clinical standpoint. These difficulties include the inability to discern region-specific alternations in permeability and confounding effects from variations in gastric emptying, intestinal transit time, microbiota-epithelial interactions, epithelial perfusion, and patient/participant non-compliance with required fasting (Galipeau and Verdu, 2016; González-González et al., 2018; Schoultz and Keita, 2020). In contrast, *in vitro* and *ex vivo* permeability assays are often better suited for mechanistic studies but may require comparatively more invasive methods (e.g., Ussing chambers), or may be limited in translatability (e.g., Caco-2 monocultures) (Galipaeu and Verdu, 2016). Importantly, intestinal barrier function, broadly, can be affected by a multitude of factors in addition to intestinal barrier permeability (or epithelial barrier permeability, more specifically) including the luminal microenvironment, mucosal-associated microbiota, and alterations in tight junction proteins (Pastorelli et al., 2013; Michielan and D’Incá, 2015). As such, mechanistic studies are crucial to tease out the individual effects of each of these contributing factors for understanding disease pathogenesis, conducting drug transport studies, and assessing preclinical efficacy and safety of novel therapeutics. Organoids are an attractive solution which overcome many of the translation barriers with traditional *in vitro* methods.

Recent application of intestinal organoid cultures includes the *in vitro* evaluation of intestinal epithelial permeability. In these studies, paracellular macromolecule (e.g., FITC-dextran) permeability is appraised following either 1) direct microinjection (Hill et al., 2017; Williamson et al., 2018); or 2) creation of a serosal-luminal concentration gradient to concentrate macromolecule in the luminal space of each organoid (Pearce et al., 2018; Bardenbacher et al., 2019; Xu et al., 2021a; Xu et al., 2021b). Due to the mechanistic nature of these evaluations, researchers have been able to link changes in epithelial permeability with causative cellular factors under controlled conditions. For example, Rallabandi et al. (2020) showed that after initial absorption of FITC-dextran into the luminal space, luminal FITC-dextran concentration decreased only marginally over the next 30 min interval due to recapitulated *in vivo* GI absorption properties, thus accurately mimicking an *in vivo* model. Further, separate studies from Xu et al. (2021a) using CD patient-derived organoids linked patient clinical measures with organoid inflammatory status and epithelial permeability. Although no differences in baseline epithelial permeability were seen between organoids from CD patients and healthy controls, the addition of proinflammatory cytokines (i.e., TNF-α, IFN-γ, and IL-1β at 20 ng/ml) to culture media caused markedly increased epithelial permeability in CD-organoids compared to respective, unchallenged controls. Conversely, addition of corticosteroid (i.e., 10 μM prednisolone) to the culture media attenuated this response to near baseline levels (Xu et al., 2021b). These findings are consistent with results obtained by d’Aldebert et al. (2020). Further, IFN-γ exposure stimulated increased expression of IL-28A in CD-organoid cultures, mimicking elevations in IL-28A seen in both plasma and biopsy samples from paired patient controls (Xu et al., 2021a). Collectively, these studies demonstrate physiological- and pharmacological-responsiveness reflective of *in vivo*, patient-specific, IBD-associated pathology.

Adult stem cells within intestinal crypts undergo differentiation along the crypt-villus axis into different cell lineages including enterocytes, Paneth cells (enteroids only), goblet cells, and enteroendocrine cells. (Mariadason et al., 2005; Stegmann et al., 2006); The relative populations of these cell types differ in a regional-specific manner, reflective of the functional specialization of each individual intestinal segment (e.g., duodenum, jejunum, ileum, etc.), or due to disease-related alterations (e.g., CD and UC) (Comelli et al., 2009). Likewise, retention of these location- or disease-specific gene expression profiles within long-term intestinal epithelial organoid cultures were first characterized by Middendorp et al. (2014) and Dekkers et al. (2013), respectively. In humans, IBD-patient derived organoids retained several inflammatory features in culture including epithelial pseudo-stratification, slow growth, reduced viability/metabolic activity status, and alterations in tight-junction proteins. Similarly, incubation of control (IBD-unaffected) organoids with a proinflammatory cocktail (i.e., TNF-α, IL-1, IL-6) induced a similar phenotype to affected organoids; including proinflammatory chemokine overexpression, decreased expression of TJ proteins, reduced cell viability, and alterations in cell populations reflective of a highly proliferative state (d’Aldebert et al., 2020). Similarly, preliminary comparison of canine patient-derived organoids showed stratification between IBD-affected and control groups for Lgr5+, ALP, PAS, NeuroG3, Zo-1, and Ki-67, phenotype markers of intestinal stem cells, enterocytes, goblet cells, enteroendocrine cells, zonulin-1 tight junction protein, and cell proliferation, respectively (Kurr et al., 2020). In contrast, significant exogenous and/or genetic measures are required to induce a simplified, IBD-like disease in mice that fails to model the chronic, multifaceted nature of IBD pathophysiology (DeVoss and Diehl, 2014).

Organoids also provide an opportunity to further evaluate the effect of genetics on intestinal health. As genetic mechanisms contributing to the development of cIBD are identified, they can be edited or removed from the cell lineage *via* adenoviral transduction, as has been previously demonstrated using the organoid technology (Stewart et al., 2021). This will allow for assessment of the organoid function with and without genetic influence. Furthermore, the interplay between intestinal epithelial cells and the immune system can be evaluated using co-cultures of organoids and immune components, such as innate immune cells (Ihara et al., 2018; Staab et al., 2020) and dendritic cells (Sebrell et al., 2019).

Canine intestinal organoids are well-developed, characterized and used for translational research (Mochel et al., 2017; Chandra et al., 2019; Ambrosini et al., 2020b; Kramer et al., 2020). Canine organoids have been shown to present relevant cell differentiation and structure, as assessed by light and Transmission Electron Microscopy and RNA *in situ* hybridization (Chandra et al 2019). Of particular relevance, canine organoids were noted to harbor tight junction proteins, Paneth cells, enteroendocrine cells, adult intestinal stem cells, stem cell progenitors and Tuft cells (Chandra et al., 2019). Furthermore, expression of pro-inflammatory cytokines and antimicrobial peptides produced by Paneth cells were also identified. In addition to structural and compositional relevance, functionality was also demonstrated by these authors (Mochel et al., 2017; Chandra et al., 2019; Ambrosini et al., 2020a). In this same study, canine organoids demonstrated metabolic activity during differentiation, functional cystic fibrosis transmembrane conductive regulator (CFTR) chloride channels and uptake of exosome-like vesicles secreted by the parasite *Ascaris suum* (Chandra et al., 2019). Canine intestinal organoids from dogs with cIBD present a unique opportunity to further utilize a large animal model of spontaneously occurring IBD while still reducing and refining the use of terminal or invasive live animal studies. Use of organoids will expedite screening of drugs that target the epithelial components of intestinal diseases which will promote safer and more efficacious therapies in an accelerated timeframe (Foulke-Abel et al., 2014). Continued development and utilization of a bioarchive of intestinal organoids from dogs with cIBD (Chandra et al., 2019) enhances the ability for translational scientists to capitalize on the multifactorial nature of this spontaneous model of IBD, while retaining the efficiency of an *ex vivo* model. The ability to use canine organoids from a spontaneously occurring model of disease (i.e., dogs with cIBD) presents a unique opportunity for “dual screening” of treatments in a spontaneous animal model prior to formal testing in human clinical trials.

## Current Research Directions for Optimization of Organoids Technology

Although organoid systems allow for accurate recapitulation of the host biology, they may have limited applicability in studies evaluating nutrient and drug transport or host-microbiome interactions. In fact, polarization of the intestinal epithelium means that nutrient and/or drug transporters on the apical membrane are enclosed within the organoid lumen and difficult to access (Zietek et al., 2015). To tackle this issue, organoid-derived epithelial monolayers (e.g., in Transwell culture) have garnered recent attention as clinically relevant *in vitro* models of the intestinal barrier function (Kozuka et al., 2017; van Dooremalen et al., 2021). Compared to traditional culture systems, preliminary studies show that monolayer cultures do not induce any significant changes in gene expression profiles or activation of transcription factors of the intestinal organoids (Takahashi et al., 2021), demonstrating the expected suitability of this model. Further, these applied systems may have added applicability for nutrient and drug transport studies (Zietek et al., 2015; Xu et al., 2018; Zietek et al., 2020) with validated methodology published for both canine (Ambrosini et al., 2020b) and human (Kozuka et al., 2017) cultures. However, use of monolayers derived from organoids requires further evaluation and careful monitoring to ensure that the same desirable characteristics obtained in organoids (cell differentiation, structure and function) are preserved. Microfluidics-based models (i.e., organoid-on-a-chip) have further advanced the physiological relevance on these techniques by replicating spatiotemporal changes in chemical and mechanical cues within the intestinal lumen (Kim et al., 2017; Velasco et al., 2020), increase throughput of culturing techniques, and have facilitated organoid use in co-cultures with cellular (e.g., immune) or bacterial populations (Min et al., 2020). This represents a unique opportunity for continued development and exploration of candidate treatments for this debilitating life-long disease.

## Conclusion

In summary, IBD is a serious debilitating disease that has remained challenging to effectively study due to its multifactorial nature. The prevalence of IBD is rapidly increasing and likely to continue given its association with Westernized modern lifestyles. Demonstration of drug efficacy and safety in animals has been and remains the best way to gain sufficient experience to initiate ethically designed human trials. While studies in mice have been pivotal in advancing genetic and cellular discoveries, murine models often lack key clinical signs or temporal pathological changes representative of IBD. Specifically, some limitations of mouse models are associated with the use of genetic knockouts to induce immune deficiency and disease. This is vastly different from the natural course of IBD developing in immunologically competent organisms, as is the case in humans and dogs. This was recently exemplified by the failures of the anti-IL17/IL13/IL10 candidate drugs in IBD clinical trials. The success of therapeutic approaches based on stem cells requires an improvement of animal disease models to recapitulate human phenotypes more faithfully, including the use of animals that have organs comparable in size and physiology to those of humans. The utilization of canine intestinal organoids, an *ex vivo* model, to improve the study of IBD/cIBD will improve efficiency and welfare concerns commonly associated with *in vivo* models of disease. Together, this presents a pathway forward for improved pharmaceutical discovery and treatment options for patients with IBD and cIBD.
